# An Account of the Post Mortem Examination of the Late Hon. Daniel Webster

**Published:** 1853-03

**Authors:** J. B. S. Jackson

**Affiliations:** Prof. of Pathology in Harvard University


From the American Journal of Medicine.

*An Account of the Post Mortem Examination of the late Hon. Daniel Webster.* By J. B. S. Jackson, M. D., Prof. of Pathology in Harvard University The emaciation was very marked, as shown by the state of the integuments and muscles; the latter being wasted, pale and flabby.

*Abdomen.*—The peritoneal cavity contained eleven pints of serum. There were also old and strong adhesions about the spleen, the gall-bladder, the cœcum, and to a small extent between the left extremities of the arch of the colon and the parietes of the abdomen. The stomach was distended, and contained half a pint of very dark blood, about one half of which was in the state of a soft coagulum; and this was the only appearance that was found of coagulum in any part of the body. The mucous membrane was deeply stained by the contents, generally rather soft, and in the pyloric portion somewhat mamellonated. The intestines were opened throughout, washed, and fully examined with reference to the diarrhœa that had so long existed. Blood was found throughout in very considerable quantity as far as the descending colon, below which there was no trace of it: in the large intestine it was altered as usual in color. Mucous membrane stained by the contents so far as blood extended. In the large intestine were numerous herniæ of the mucous membrane, so common in this situation; from many of these, small masses of fæces or of mucus could be forced out, and these were the only traces of fæces that were found. Otherwise, the mucus membrane of the intestines appeared quite healthy; there being nowhere any ulceration to explain the diarrhœa, or ecchymosis connected with the hemorrhage. The liver was, throughout, very markedly granulated; dense, and contracted in size; the color externally was greenish or bronzed, but internally everywhere of a pale red, showing, as we may not unfrequently observe, the inappropriateness of the term "cirrhosis," which would generally have been applied to the present case. Weight of the organ, three pounds and one-third, avordupois. Bile in the gall-bladder nearly black, and of a very tarry consistence. Spleen small, pale, and shrivelled. Investing membrane to some extent opaque, white, thickened and condensed; this change being probably due to the old peritoneal affection. Kidneys and pelvic organs healthy.

*Thorax.*—Old pleural adhesions over nearly the whole of the right side; none on the left. Lower lobe of the left lung, and the two lower lobes of the right much congested, and very dark; a change that undoubtedly occurred toward the close of life, being simply passive. Heart flacid; very little blood in cavities, and this was quite liquid. Slight disease of aortal valves, but organ otherwise healthy. Foramen ovale; a small vavular opening existed. Aorta not ossified, except to a small extent in the abdomen.

*Head.*—The membranes of the brain were most remarkably diseased. In the cavity of the arachnoid was a layer of fibrine, which covered almost entirely and about equally the convexity of both hemispheres; it did not extend, however, beneath or between them, nor about the cerebellum. In the recent state, it had a rather dull, yellowish, infiltrated, œdematous appearance, being one-fourth of an inch in thickness over the upper surface, but becoming gradually more thin on the sides, where it terminated in a thin edge. The adhesion to the dura mater was in some parts quite close; but it was generally very readily stripped off, and left the arachnoid with its usual polish. It was more adherent to the sub-adjacent membrane, this last being irregular, and having generally a clouded and slightly opaque appearance, with many milk-white spots, but without any appreciable thickening. The quantity of serous effusion into the membranes was altogether large. The subarachnoid tissue corresponding to the layer of fibrine above described, was infiltrated with a straw-colored serum in some places, separating the convolutions from each other; this separation was quite remarkable at the posterior part of the right cerebral hemisphere on its upper surface and near the median line, there being also a slight depression at this part. The dura mater adhered firmly to the calvaria, but was healthy in structure, as were the membranes otherwise; there was, however, a serous infiltration into each plexus choroides; though no more, if not less than usual, into the lateral ventricles. No appearance of recent meningitis, and no effused blood or cysts in or about the false membrane. The brain itself was perfectly healthy, and the arteries at the base very nearly so. Cranium healthy. Over the right frontal region a scar existed, the result of the injury that occurred last May; integuments not otherwise remarkable. A portion of the fibrine from the arachnoid cavity having been removed for microscopical examination, it was found, some hours afterwards, and when the serum with which it had been infiltrated was absorbed, to have almost the consistence of one of the natural tissues of the body, being strong enough to bear considerable traction; it also appeared, then, to have somewhat of a laminated structure, and blood-vessels were distinctly seen in it with the naked eye. Dr. Wyman found it "organized, and, in some places, vascular. Under the microscope, the lymph was resolved into minute fibres, like those forming the white fibrous element of areolar tissue, and including in their meshes large numbers of minute granules."

Recapitulating the points of interest in this case, it will be observed that the immediate cause of death was hemorrhage from the stomach and bowels. For this no source could be found in the lesion of any vessel; it must, therefore, be regarded as a simple exhalation, dependent upon a disorganization of this fluid, indicated, moreover, by the almost entire absence of coagulation. The relation of this hemorrhage to the disease of the liver will also be noted as coinciding with previous experience; it being well known, that, in certain cases where there is an altered action of this organ, there is a tendency to disorganization of the blood, manifesting itself thus in hemorrhage.

The morbid appearances observed in the cerebral membranes possess, also, very great interest in several aspects. It will be unnecessary to dwell upon the particular appearances carefully described above. A very full and clear description of these interesting forms of extravasation has been published by Mr. Prescott Hewitt. in the twenty-eighth vol. *Medico-Chirurgical Transactions of London*, and the appearances, in this case, coincide with those there described. Grisolle (*Pathologie Interne*, vol. i.) has also well described this affection, after the original descriptions of Serres, Baillarger, Boudet, and Prus, who were the first to call attention to this particular lesion. The case of Mr. Webster may be regarded as unique, however, in this respect, that no impairment of the power of the nervous system was observed before death: for although a few symptoms, such as his mode of locomotion, his sense of falling, and a slight hesitation of his speech, may now be remembered and connected with this condition, it will be sufficient to prove the entire absence of any suspicions of the kind during life to state that the brain would not have been examined at the autopsy, except for the desire of making the measurements, &c., recorded below. The connection of this meningeal hemorrhage with the cirrhus of the liver will also give rise to interesting speculation; for although it is quite probable that the origin of the effusion should be ascribed to the accident in May, still, it is not unlikely to be remotely dependent upon the disorganization of the blood consequent upon the disease of the liver, since among Mr. Hewitt's cases there are some recorded where an effusion quite equal to this took place in connection with a cirrhus, without any injury at all. It is possible, moreover, that the accident may not have been the cause of the effusion, which may have taken place since that time; but, in the presence of what would appear an adequate cause, it will be unnecessary to look beyond.

In the treatment of the disease, attention was particularly directed to the duodenal obstruction, relief from which was obtained by the laxatives occasionally administered, and these, with opiates, were almost the only important medical agents.

The following very interesting account of the cranial cavity and brain, is furnished by Dr. Jeffries Wyman: The dimensions of the brain, as indicated by the measurements of the cranial cavity, were as follows:—Longitudinal diameter, 7⅞ inches; transverse diameter, 5¾; vertical, 5½; breadth of occipital fossa, 4¾; breadth of frontal fossa, 5. The posterio rclinoid processes were seven-eighths of an inch in front of the centre of the cranial cavity.

The circumference of the head was 23¾ inches, and the distance from the meatus of one ear to that of the other, over the top of the head was 15 inches. The capacity of the cranium, determined according to the method adopted by the late Dr. S. G. Morton, of Philadelphia, was 122 (one hundred and twenty-two) cubic inches.

The substance of the brain was firm to the touch, and, as regards color and consistence, appeared to be healthy. The depth of the spaces between the convolutions was, on the vortex, seven eights of an inch, and the "cortical" or gray substance, was three sixteenths of an inch in thickness. The corpus callosum, or the great cerebral commissure, was large, measured four inches in length from before backwards and at the central portion was one-fourth of an inch in thickness. The pineal body, as in the great majority of instances, contained calcareous concretions.

The weight of the brain, including the cerebrum, cerebellum, and medulla oblongata, as far as the lower extremity of the pyramids, was (in avoirdupois):

| | Lbs. | Oz. | Dra'ms. | Grs. | Grains. |
|---|---|---|---|---|---|
| Brain (encephalon) | 3 | 5 | 8 | 17.75= | 23,424.0 |
| Cerebrum - - | 2 | 14 | 7 | 14.09= | 20,330.4 |

The measurements which have been given above are, almost without exception, of unusual proportions. The average length of the cranial cavity does not exceed six and a half inches; its transverse diameter is five inches, and the vertical a little less.

The cranial capacity was very unusual, the largest which has yet been recorded, though measurements in cubic inches have as yet been made by comparatively few observers. In Dr. Morton's tables of the measurements of 623 crania of different nations, including Caucasians, Mongolians, Malays, Americans and Negroes, only four instances occur in which the capacity exceeded one hundred cubic inches; of these the largest were one English skull, measuring 105, and one German, 114 cubic inches. According to Dr. Morton, the average capacity for the Teutonic family (including English, Germans, and Anglo-Americans) is 92 inches.

The two superficial measurements of the head were very nearly those of Cuvier, the circumference of whose head was 22 inches 4 lines (French,) and the measurement from ear to ear over the top was 15 inches. The circumference of Napoleon's head is reported to have been 23 inches.

The weight of the brain deviated much less from the average than the measurements ; it was entirely out of proportion to the unusual dimensions of the cranial cavity. The average weight of an adult healthy male brain is 49½ ounces, or three pounds 1½ ounces avoirdupois, As has been already stated, there existed an effusion of serum into the subarachnoid areolar tissue, and of serum and lymph into the arachnoid cavity. The lymph had existed for a long time ; it covered the convex surface of the cerebral lobes, was a quarter of an inch in its thickest portion, and extended to the sides, where it became quite thin. Both serum and lymph, there can be no doubt, encroached upon and occupied the space once filled with cerebral substance. The weight given above, therefore, cannot be regarded as being equal to the weight of the brain in a state of health. This last we now have no means of determining except by an approximation, which has been made in the following manner, in accordance with a suggestion by Professor Treadwell, of Cambridge.

The specific gravity of the brain is, according to Cruveilhier and others, 1030, water being 1000. A cubic inch of water weighs 252.5 grains, and 122 cubic inches (the cranial capacity) would equal 30.805 grains, to which must be added 3 per cent., or 924 grains, (the excess of specific weight of brain over water,) which gives 31,829 grains as the full capacity of the cranial cavity in weight for cerebral substance. The brain, however, does not actually fill the whole cavity; a correction must, therefore, be made for the spaces occupied by the tentorium, falx, sinuses, the dura mater of the calvaria, and the cephalo-spinal fluid at the base of the brain. If we deduct eight ounces for such spaces, we shall have an actual weight of 38,329 grains; or, if nine ounces are deducted, 26,891 grains. Taking the last approximation as the one the least liable to error of excess, Mr. Webster's brain will be found to rank among those whose brains are generally cited as instances of remarkable size, as follows :

| | Lbs. | Oz | Drs. | Grs. | Grs | Oz |
|---|---|---|---|---|---|---|
| Cuvier, | 4 | 0 | 5 | 10 = | 28,147 = | 64⅓ |
| Webster, | 3 | 15 | 12 | 0 = | 27,891 = | 63¾ |
| Abercrombie, | 3 | 15 | 0 | 0 = | 27,562 = | 63 |
| Spurzheim, | 3 | 7 | 1 | 0 = | 24,089 = | 55 1-16 |
| Dupuytren, | 3 | 1 | 10 | 27 = | 21,738 = | 49 11-16 |

The brains, the weights of which (in avoirdupois) are included in this table, are not the only ones on record remarkable for size. In the table of Dr. Sharpey, already quoted, there are enumerated as weighing between 55 and 59 ounces, avoirdupois, inclusive, 28 brains ; and between 60 and 65 ounces, 7. Nothing is said of the individuals from whom they were taken ; of the two largest, one weighed 63 and the other 65 ounces ; it is not improbable that these were the brains of Abercrombie and Cuvier; 63 ounces being precisely the weight of the former. In making out the table, all instances with fractional parts were classed with the next integral number; and as Cuvier's brain weighed over 64 ounces, it would rank as 65 ounces. If this be not the explanation, then there is on record a larger healthy brain than that of Cuvier.

